# Machine Vision-Based Method for Estimating Lateral Slope of Structured Roads

**DOI:** 10.3390/s22051867

**Published:** 2022-02-26

**Authors:** Yunbing Yan, Haiwei Li

**Affiliations:** School of Automobile and Traffic Engineering, Wuhan University of Science and Technology, Wuhan 430065, China; 18571638149@163.com

**Keywords:** road lateral-slope estimation, machine vision, image-perspective principle, SCNN algorithm

## Abstract

Most of the studies on vehicle control and stability are based on cases of known-road lateral slope, while there are few studies on road lateral-slope estimation. In order to provide reliable information on slope parameters for subsequent studies, this paper provides a method of structured-road lateral-slope estimation based on machine vision. The relationship between the road lateral slope and the tangent slope of the lane line can be found out according to the image-perspective principle; then, the coordinates of the pre-scan point are obtained by the lane line, and the tangent slope of the lane line is used to obtain a more accurate estimation of the road lateral slope. In the implementation process, the lane-line feature information in front of the vehicle is obtained according to machine vision, the lane-line function is fitted according to an SCNN (Spatial CNN) algorithm, then the lateral slope is calculated by using the estimation formula mentioned above. Finally, the road model and vehicle model are established by Prescan software for off-line simulation. The simulation results verify the effectiveness and accuracy of the method.

## 1. Introduction

With the gradual development of vehicle intelligence, the control requirements for vehicles are becoming more and more refined. To achieve this, in addition to the need to obtain the vehicle’s own operating parameters and structural parameters, external environmental parameters are also necessary. The road slope is one of the most important parameters of the external environment, and the acquisition of this parameter is crucial for the control of the vehicle. At the same time, road curves tend to cause a high incidence of traffic accidents, and related reports indicate that the number of accidents and fatalities on curved road sections has increased year by year among traffic accidents that have occurred in recent years [1]. According to the Fatal Accident Reporting System (FARS) in the United States, the average number of roadside accidents accounts for more than 39% of fatal accidents each year. In China, about 50% of traffic accidents with more than three fatalities are roadside accidents. A European study also showed that about 20% of road traffic accidents each year are roadside accidents, while the roadside accident fatality rate is more than 35%. According to another study [2], about 1/3 of roadside accidents occur in curved sections, and small-radius curved sections are often considered as roadside-accident-prone areas [3,4]. There are many reasons for roadside accidents, such as poor road alignment design, inappropriate avoidance measures taken by drivers, inattention, or understeering of vehicles due to high speed through curved sections [5]. In order to ensure the smooth operation of vehicles on curved roads, the ultra-high transition section is often set. Generally speaking, in order to make the gradient section elevation easy to calculate and construct, road design often uses a linear gradient; however, for long, gentle curves and S-curved sections, the curve super-high-gradient way is used, which can improve road drainage performance and driving stability [6]. There are many studies on the ultra-high gradient method; the values of parameters such as the ultra-high transition method, ultra-high gradient rate, and the formula for calculating the length of the ultra-high transition section are specified in “Geometric Design of Highways and Urban Roads” and “Interpretation and Application of Japanese Highway Technical Standards”, edited by AASHTO, USA [7,8]. Jeong [9] investigated the effect of longitudinality on the drainage of ultra-high rainwater at a zero cross-section in the middle of a reverse curve for the poor characteristics of drainage. Jeong [10] established a two-dimensional finite volume diffusion model to simulate the flow field on a geometric surface, to explore the relationship between the pavement panel flow-distribution and the cross slope and longitudinal slope of the pavement, etc. Zhang [11] proposed a safe and reasonable superhigh gradient rate based on the safety boundary. Fitzpatrick [12] discussed methods concerning the determination of side-friction factors and transition lengths. Arslan [13] compared aesthetic transition curves with classical transition curves in terms of vehicle kinematics, and derived curvature and superelevation functions. The superelevation approach to highway design was evaluated considering the stability of large vehicles, focusing on the reassessment of the relationship between factors such as side-friction resistance, minimum radius and design speed. There is no unified standard for the design of the ultra-high transition section, while there is a more unified standard for the design of the ultra-high non-transition section, as shown in Equation (1):

(1) $$i_{h}+\mu =\frac{V^{2}}{127R}$$

where $i_{h}$ is the road superelevation; *μ* is the lateral force coefficient; *V* is the vehicle speed; and *R* is the road curvature. However, there are four different allocations of $i_{h}$ and *μ* [14], and different allocations are used for roads with different curvatures and different operating speeds, so a more accurate estimation of the superelevation cannot simply be made by the road curvature and the road grade.

Furthermore, there is relatively little research on lateral slope estimation [15], and most of the research on vehicle control and stability is based on cases wherein the lateral slope of the road is known, or the lateral slope is ignored. In the actual operation of a vehicle, the road conditions change according to the environment, and the lateral slope is not a constant value, which makes the actual control effect of the vehicle not optimal [16,17,18,19,20,21,22]; thus, the study of road lateral-slope estimation is necessary.

The research related to lateral-slope-angle estimation is currently divided into two main types: direct measurement with the help of sensors, and indirect measurement. The first method, affected by production cost and measurement accuracy, is still in the theoretical research stage and is not suitable for direct application to vehicles [23,24]. Therefore, most of the current studies belong to the second method, which are based on the existing sensors of the vehicle and design algorithms for slope estimation. Moreover, this method is mainly divided into two types based on kinetic estimation and kinematic estimation. Menhour [25] measured the lateral slope by designing a linear two-degree-of-freedom dynamics model with an unknown-parameter slip-film observer, but this method requires solving for cases wherein the pavement friction coefficient is known. To solve this problem, Tseng [26,27] proposed a lateral-slope-dynamics estimation method for cases of unknown pavement friction coefficient based on it; however, the method did not take into account the effect of vehicle side-inclination angle on slope estimation, thus limiting further application of the results. Therefore, Guan Xin [28] estimated the vehicle’s lateral-inclination angle based on Tseng H E using the vehicle droop position sensor information, thus decoupling the true value of the road lateral slope, but the estimation results of this method fluctuate greatly when the vehicle is driving in and out of the ramp. In order to make the model estimation performance stable and reduce the risk of model divergence, Jeong [29] designed a finite memory estimation model; however, it ignored the effect of vehicle lateral-inclination angle on slope estimation. Hyun [30] proposed a method combining Bayesian tracking and Kalman filtering to jointly estimate the road lateral-slope angle by measuring the vehicle lateral acceleration as well as the vehicle lateral-tilt angle, which can not only accurately solve the series of problems mentioned above, but also has high estimation accuracy in the case of large slope angles; however, the algorithm is complex and has high hardware requirements. The advent of machine vision has provided a new idea for road-slope estimation, and Ustunel [31] proposed a monocular camera-based method for road lateral-slope estimation with high accuracy; however, it has only been studied for curved roads with large radius of curvature. Nevertheless, ultra-high (i.e., lateral slope) is often designed to mitigate the effect of centrifugal forces on vehicle stability [14], so the estimation of lateral slope for small-curvature-radius roads is more practical in realistic situations.

In summary, there is a lack of a lateral-slope estimation methods for small-curvature-radius roads with high measurement accuracy and high efficiency. To address this problem, this paper firstly introduces a linear road as the research object for problem analysis, then finds out the method for estimating the lateral slope of a small-curvature-radius road. It finally verifies the accuracy of the algorithm by using a Prescan modeling simulation and analyzes the error of the experimental results.

## 2. Principle of Machine Vision-Based Road Lateral-Slope Estimation Algorithm

Because the direct selection of curved roads for research is relatively complex, in order to simplify the problem, this paper first selects a straight road as the object of study, through the analysis of the image projection principle, to obtain the solution formula for the lateral slope of the road; then, it introduces the curved road for problem analysis.

### 2.1. Straight Road Cross Slope Solution

**Figure 1:**
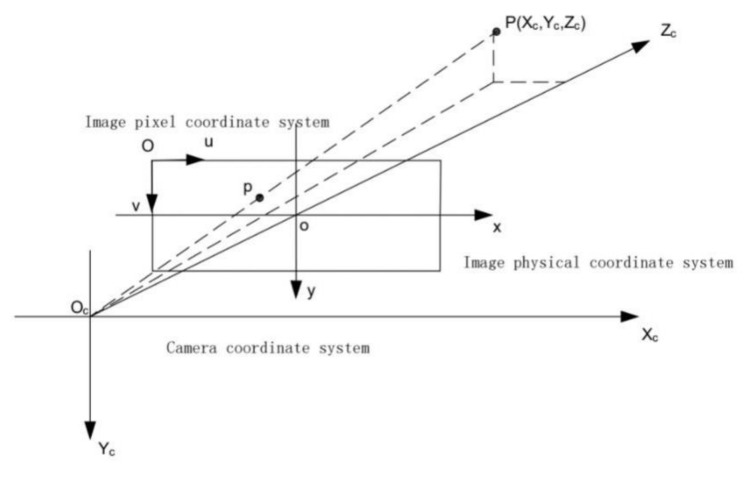
Schematic diagram of the geometric relationship between coordinate systems.

Figure 1 shows the relationship between the camera coordinate system, image physical coordinate system, and image pixel coordinate system. Where the coordinate values of the image pixel coordinate system represent the columns and rows of the image array; the image physical coordinate system is proposed to solve the drawback that the image pixel coordinate system has no physical units, and the physical units are used to represent the position of the pixel points in the image; the camera coordinate system is a three-dimensional right-angle coordinate system established with the camera optical center as the coordinate origin [32].

The transformation relationship between the camera coordinate system and the image physical coordinate system (Equation (2)), and the transformation relationship between the image physical coordinate system and the image pixel coordinate system (Equation (3)) can be obtained based on the triangular similarity [32].

(2) $$x=f\frac{X_{C}}{Z_{C}};\;y=f\frac{Y_{C}}{Z_{C}}$$

(3) $$u=\frac{x}{d_{x}}+c_{x};\;v=\frac{y}{d_{y}}+c_{y}$$

Where *x*, *y* are the two-dimensional coordinate values in the physical coordinate system of the dimensional image; *u*, *v* are the two-dimensional coordinate values in the pixel coordinate system of the image; *f* is the focal length of the camera; $X_{C}$, $Y_{C}$, $Z_{C}$ are the three-dimensional coordinate values in the coordinate system of the camera; $d_{x}$, $d_{y}$ denote the distance between pixels and pixels in the *x*, *y* direction, respectively; and $c_{x}$, $c_{y}$ are the point O in the pixel coordinate system of the image pixel coordinate system.

**Figure 2:**
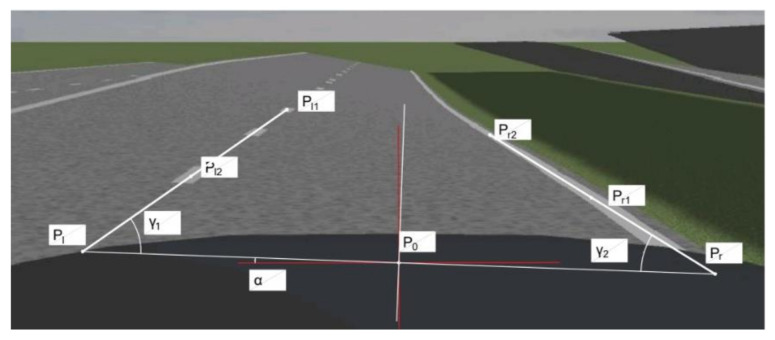
Schematic diagram of wedge-shaped pavement.

The estimation method of lateral slope is analyzed by selecting a wedge-shaped pavement as shown in Figure 2, which is a straight section of road without curves and has a lateral-slope angle α.

The figure shows the scene when the vehicle travels to the front of the ramp. The red line is the plane where the vehicle is located, and the white solid line is the plane where the wedge-shaped road surface is located. In the figure, $P_{l1}$, $P_{r1}$ and $P_{l2}$, $P_{r2}$ are the intersection points of the two-dimensional image pre-scanning lines, $L_{1}$, $L_{2}$, and the left and right lane lines, respectively, and $L_{1}$, $L_{2}$ are the horizontal straight lines at 1/4 and 1/2 of the image, respectively. $\gamma_{1}$, $\gamma_{2}$ are the angles of $P_{l1}P_{l2}$, $P_{r1}P_{r2}$ on the image and the horizontal direction, respectively; α is the road cross-slope angle; $P_{0}P_{l}$, $P_{0}P_{r}$ are the distances $d_{l}$, $d_{r}$ from the vehicle to the left and right lane lines, respectively.

Therefore, the world coordinate value of the pre-sighting point is shown in Equation (4). This world coordinate system takes the camera optical center as the origin; the *Z*-axis is the same as the camera optical axis and the vehicle forward direction; the *X*-axis is the horizontal direction perpendicular to the direction of the vehicle; and the *Y*-axis is the vertical direction perpendicular to the forward direction of the vehicle.

(4) $$\begin{array}{l} P_{l1}\;=\;\left(X_{l1},Y_{l1},Z_{l1}\right)\;=\;\left(-d_{l}\cos\left(\alpha \right),-d_{l}\sin\left(\alpha \right)-Y_{0},Z_{l1}\right)\\ P_{l2}\;=\;\left(X_{l2},Y_{l2},Z_{l2}\right)\;=\;\left(-d_{l}\cos\left(\alpha \right),-d_{l}\sin\left(\alpha \right)-Y_{0},Z_{l2}\right)\\ P_{r1}\;=\;\left(X_{r1},Y_{r1},Z_{r1}\right)\;=\;\left(d_{r}\cos\left(\alpha \right),d_{r}\sin\left(\alpha \right)-Y_{0},Z_{r1}\right)\\ P_{r2}\;=\;\left(X_{r2},Y_{r2},Z_{r2}\right)\;=\;\left(d_{r}\cos\left(\alpha \right),d_{r}\sin\left(\alpha \right)-Y_{0},Z_{r2}\right) \end{array}$$

where, $Y_{0}$ is the distance of the camera from where it is located to the horizontal ground; $X_{ji},$ $Y_{ji}$, $Z_{ji}\;$(*j* = l, *r*; *i* = 1, 2) are the three-dimensional coordinate values of the pre-sighting point under the world coordinate axis, respectively.

The physical coordinates of its projection in the two-dimensional image can be obtained by combining Equations (2) and (4) as:

(5) $$\begin{array}{l} p_{l1}\;=\;\left(x_{l1},y_{l1}\right)\;=\;\left(\frac{f\cdot X_{l1}}{Z_{l1}},\frac{f\cdot Y_{l1}}{Z_{l1}}\right)\\ p_{l2}\;=\;\left(x_{l2},y_{l2}\right)\;=\;\left(\frac{f\cdot X_{l2}}{Z_{l2}},\frac{f\cdot Y_{l2}}{Z_{l2}}\right)\\ p_{r1}\;=\;\left(x_{r1},y_{r1}\right)\;=\;\left(\frac{f\cdot X_{r1}}{Z_{r1}},\frac{f\cdot Y_{r1}}{Z_{r1}}\right)\\ p_{r2}\;=\;\left(x_{r2},y_{r2}\right)\;=\;\left(\frac{f\cdot X_{r2}}{Z_{r2}},\frac{f\cdot Y_{r2}}{Z_{r2}}\right) \end{array}$$

where, $x_{ji},$,$y_{ji}$ (*j* = l, *r*; *i* = 1, 2) are the two-dimensional coordinate values of the pre-sighted points in the physical coordinate system, respectively.

The slopes $K_{l}$ and $K_{r}$ of $P_{l1}P_{l2}$ and $P_{r1}P_{r2}$ can be obtained from the association of Equation (4) with Equation (5) as:

(6) $$K_{l}=\tan\left(\gamma_{1}\right)\;=\;\frac{y_{l2}-y_{l1}}{x_{l2}-x_{l1}}\approx \frac{Y_{l}}{X_{l}}\;=\;\frac{Y_{0}+d_{l}\sin\left(\alpha \right)}{d_{l}\cos\left(\alpha \right)}$$

(7) $$K_{r}=\tan\left(\gamma_{2}\right)\;=\;\frac{y_{r2}-y_{r1}}{x_{r2}-x_{r1}}\approx \frac{Y_{r}}{X_{r}}=\frac{Y_{0}+d_{r}\sin\left(\alpha \right)}{d_{r}\cos\left(\alpha \right)}$$

Combining Equation (6) with Equation (7) and eliminating the focal length f and the *Z*-axis coordinate value, Equation (8) can be obtained:

(8) $$\alpha =\arcsin\left(\frac{Y_{0}\left(K_{l}d_{l}-K_{r}d_{r}\right)}{\left(K_{l}+K_{r}\right)d_{l}d_{r}}\right)$$

### 2.2. Curved Road Cross Slope Solution

**Figure 3:**
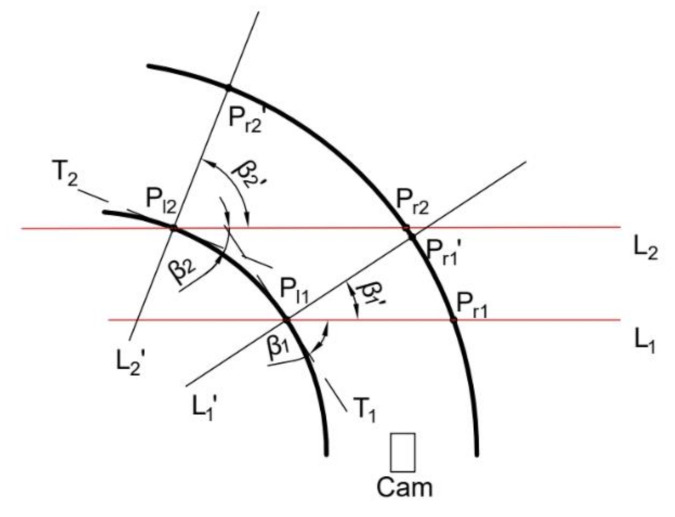
Overhead diagram of curved road section.

After the analysis in the previous section, the solution formula (8) for the cross-slope of the straight road section is obtained. When the vehicle is in the curved road section as shown in Figure 3—if the pre-sighting point is selected as $L_{1}$, $L_{2}$ and the intersection of the lane line $P_{l1}$, $P_{r1}$ and $P_{l2}$, $P_{r2}$, to estimate the cross slope angle—it is obvious that the result obtained is not the cross-slope angle of the vehicle passing through the road section.

Assuming the inclination angles $\beta_{1}$ and $\beta_{2}$ of the lane-line tangents are $T_{1}$ and $T_{2}$ at $P_{l1}$ and $P_{l2}$, then $\beta_{1}^{\prime }$ and $\beta_{2}^{\prime }$ are:

(9) β1′=π2−β1β2′=π2−β2

Rotating $L_{1}$ and $L_{2}$ by $\beta_{1}^{\prime }$ and $\beta_{2}^{\prime }$ angles, the expressions for the functions $L_{1}^{\prime }$ and $L_{2}^{\prime }$ are obtained as follows:

(10) $$\begin{array}{l} L_{1}^{\prime }:y=\tan\beta_{1}^{\prime }x+y_{1}-\tan\beta_{1}^{\prime }x_{1}\\ L_{2}^{\prime }:y=\tan\beta_{2}^{\prime }x+y_{2}-\tan\beta_{2}^{\prime }x_{2} \end{array}$$

Equation (10) and the lane-line function can be combined to obtain the coordinate values of the intersection point $P_{r1}^{\prime }$, $P_{r2}^{\prime }$ of the rotated pre-sight line $L_{1}^{\prime }$, $L_{2}^{\prime }$, and the other side of the lane line, at this time. Coordinating the $P_{l1}$, $P_{l2}$ and $P_{r1}^{\prime }$, $P_{r2}^{\prime }$ values into Equations (6)–(8) can obtain the curved-road cross-slope angle. When the road is a right turn the principle is the same, and does not need to be repeated.

## 3. Estimation of the Position of the Vehicle Relative to the Lane Line and the Slope of the Lane Line Tangent

Through the above principle analysis, the relationship equation between road slope and $d_{l}$, $d_{r}$ and the coordinates of auxiliary points is obtained. In practical application, it is also necessary to identify the lane lines based on vision and fit the lane lines to obtain the parameters of lane line function, $d_{l}$, $d_{r}$, and lane-line tangent slope. In general, structured roads have more obvious lane-marker lines, which are easy to identify and estimate. For this reason, this paper takes structured roads as an example to illustrate.

### 3.1. Lane Line Detection Algorithm

**Figure 4:**
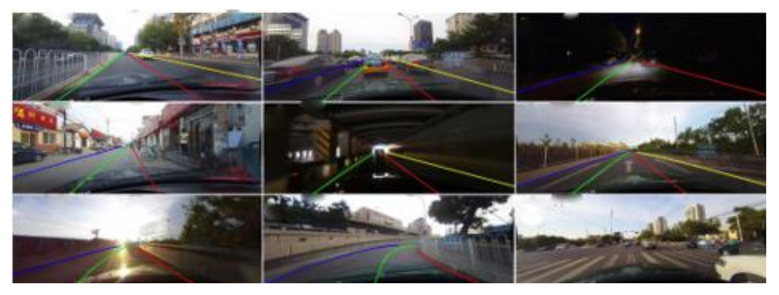
Effect of SCNN lane line detection algorithm.

In this paper, the SCNN (Spatial CNN) lane-line detection algorithm [33] is selected to obtain the lane line fitting function. SCNN is a new neural network structure for extracting lane lines. Compared with the traditional neural network that convolves directly between the layers, the net convolves in a certain direction and order. This neural network algorithm has the advantages of higher computational efficiency and faster training speed. Figure 4 shows the effect of lane line detection in various environments.

The lane line fitting function can be obtained by extracting and fitting the lane lines by the above algorithm as shown in Equation (11):

(11) $$\begin{array}{l} B_{k,1}(u)\;=\;\left\{\begin{array}{c} 1,\;u_{k}\leq u\leq u_{k+1}\\ 0,\;\mathrm{other}\; \end{array}\right.\\ B_{k,d}(u)=\frac{u-u_{k}}{u_{k+d-1}-u_{k}}B_{k,d-1}(u)+\frac{u_{k+d}-u}{u_{k+d}-u_{k+1}}B_{k+1,d-1} \end{array}$$

where *u* is the control node; *d* is related to the number of functions, which is taken as four in this paper; and n is the number of control points.

### 3.2. Estimation of Vehicle Position Relative to the Lane Line

**Figure 5:**
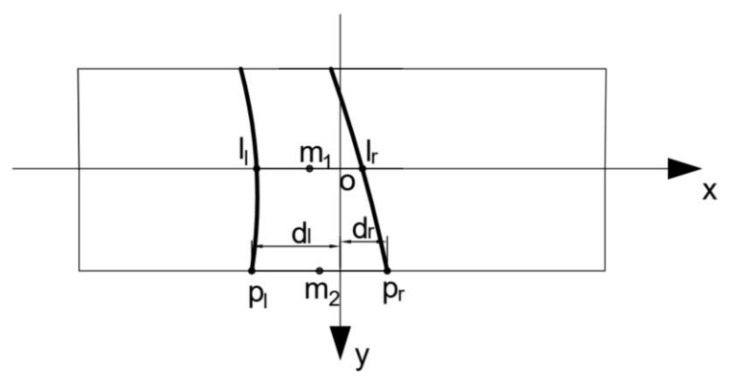
Diagram of lane line projection.

From Equation (8), we need to know $d_{l}$, $d_{r}$. According to the lane line fitting function obtained in the previous section, we can obtain the intersection of the left and right lane lines with the bottom of the image as $p_{l}=\left(x_{l},y\right)$ and $p_{r}=\left(x_{r},y\right)$ in Figure 5, respectively. Since the general lane deviation angle is not too large when the vehicle is in motion, $p_{l}p_{r}$ can be regarded as the projection of the lane-line width on the image, leading to Equation (12):

(12) $$d=\frac{Z_{\min}}{f}\left|p_{l}p_{r}\right|$$

where *d* is the width of the lane line; $Z_{min}$ is where the camera can capture the nearest place and horizontal distance to the camera optical center; *f* is the focal length of the camera; and $p_{l}p_{r}$ $p_{l}$ and $p_{r}$.

The projection of the lane line in the image captured by the camera is shown schematically in Figure 5.

From the figure, as well as Equations (12) and (13) is obtained:

(13) $$\frac{\left|x_{l}\right|}{\left|x_{r}\right|}=\frac{d_{l}}{d_{r}};\;d_{l}+d_{r}=d$$

### 3.3. Estimation of Lane Line Tangent Slope

According to Equations (9) and (10), it is necessary to know the tangent slope of the curved lane, and due to the camera viewpoint, the 3D information such as the curvature of the lane line cannot be obtained directly from the original image, so this paper derives the method of lane-line tangent-slope estimation from the principle of inverse perspective.

A point in the world coordinate system is mapped onto the pixel plane, a process called perspective transformation [18], as shown in Equation (14):

(14) $$Z_{C}\left[\begin{array}{c} u\\ v\\ 1 \end{array}\right]=M_{1}M_{2}\left[\begin{array}{c} X_{W}\\ Y_{W}\\ Z_{W}\\ 1 \end{array}\right]=MX_{W}$$

where $Z_{C}$ is the value of the *Z*-axis coordinates of the point in the camera coordinate system, which can be seen as a loss of dimensionality in the process of perspective. Inverse perspective will be taken as 1, where $M_{1}$ is only related to the structure of the camera, called the internal parameters of the camera (internal parameters); $M_{2}$ is only related to the position of the camera in the world coordinate system, called the external parameters of the camera (external parameters); and *M* is a 3 × 4 projection matrix.

Thus, the image coordinates can be transformed into world coordinates by using Equation (14), which allows:

(15) $$Z_{C}M^{-1}\left[\begin{array}{c} u\\ v\\ 1 \end{array}\right]=\left[\begin{array}{c} X_{W}\\ Y_{W}\\ Z_{W} \end{array}\right]$$

In Figure 5, $m_{1}$ and $m_{2}$ are the midpoints of $l_{l}l_{r}$ and $p_{1}p_{2}$, respectively. Then, their corresponding points, $M_{1}\left(X_{1},Y_{1}\right)$, $M_{2}\left(X_{2},Y_{2}\right)$, in world coordinates can be solved by Equation (15); the slope *K* of a line, $M_{1}M_{2},\;$ determined by these two points is:

(16) $$K=\frac{X_{1}-X_{2}}{Y_{1}-Y_{2}}$$

For conventional roads, the curvature transformation is relatively gentle, so it can be assumed that the slope of the corresponding lane-line tangent at the midpoint of $M_{1}M_{2}$ is *K*.

## 4. Simulation Experiment Verification and Analysis

The paper builds several road models with different slopes and curvatures using Prescan software, and places the vehicle on different points in the lane. By setting the camera sensor at 1.2 m above the vehicle from the ground, we can obtain the image of the structured road ahead and use it to conduct offline simulation experiments.

### 4.1. $d_{l}$ and $d_{r}$ Estimation Validation Experiments

**Figure 6:**
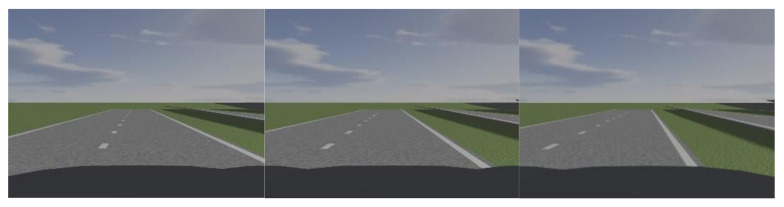
Schematic diagram of the experimental part of PRESCAN $d_{l}$ and $d_{r}$ estimation.

In order to verify the accuracy of the vehicle’s relative-position-estimation algorithm, the vehicles are placed at seven points on the lane octant for estimation verification experiments. Figure 6 shows the modeling diagram of the simulation experiment, and Table 1 shows the experimental results.

The absolute value of the estimated error value is less than 0.1 m, and the estimated value is more accurate. The analysis of the experimental results shows that the cause of this error is due to visual bias. To further improve the accuracy of the estimated value, and consequently improve the accuracy of the subsequent estimation, an artificial parameter ε is introduced, which is obtained by fitting the function of the error value to the 2D image value $\left|x_{l}\right|/p_{l}p_{r}$, so that the final estimated value ${\bar{D}}_{l}$ and *ε* as shown in Equation (17):

(17) x=xlplprε=−1.264x3+2.059x2−1.102x+0.1923Dl¯=Dl−ε

**Figure 7:**
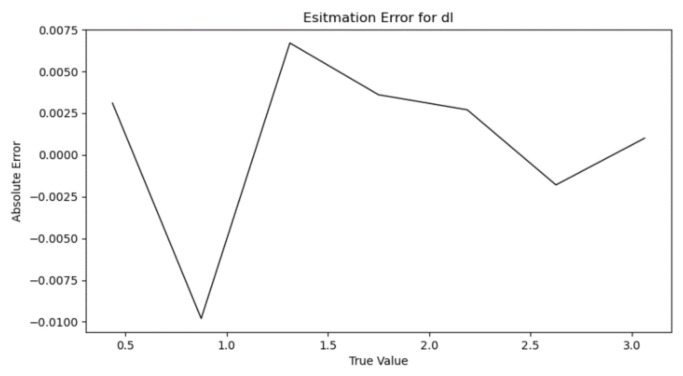
Plot of experimental results of $d_{l}\;$ estimation.

After introducing the parameter ε, the vehicle is randomly placed at any position of the lane line, and the $d_{l}$ estimation results are shown in Figure 7.

From Figure 7, it can be seen that the error is controlled within 0.01 after introducing the artificial parameter ε, which satisfies the accuracy requirement of the subsequent algorithm for $d_{l}$.

### 4.2. Verification Experiments of Lane Line Tangent Slope Estimation

**Figure 8:**
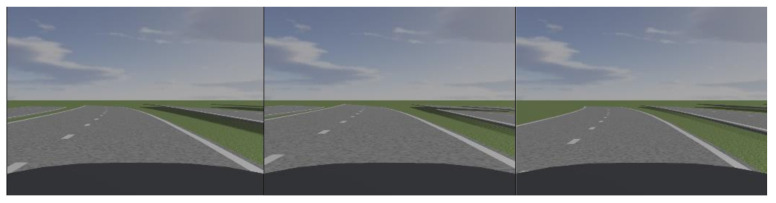
Schematic diagram of the experimental part of Prescan lane-line tangent-slope estimation.

**Figure 9:**
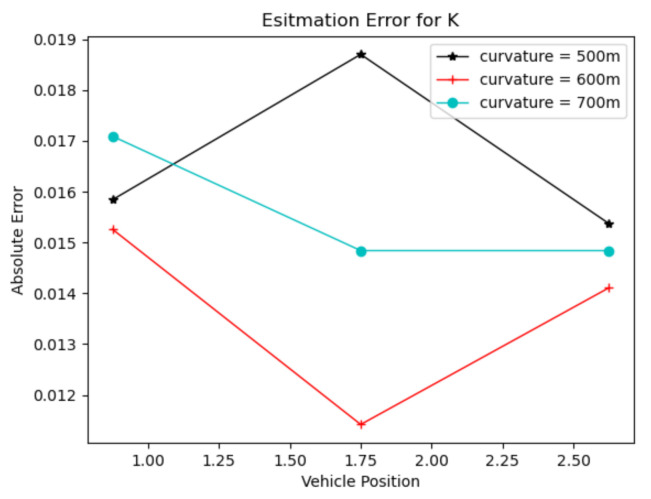
Graph of lane-line tangent-slope estimation results.

To verify the accuracy of the lane line tangent slope estimation algorithm, circular curves with radii of curvature of 500, 600 and 700 m were established in Prescan, as shown in Figure 8. The vehicles were placed at three points in four equal parts of the lane for verification experiments, and the experimental results are shown in Figure 9.

Since it is not easy to find the exact data point on the pixel image, this will have an impact on the subsequent estimation. It can be seen from the above figure that the closer the vehicle is to the center of the lane line, the more accurate the estimated value of K. However, the error value of K is no more than 0.02, which satisfies the requirement of the subsequent algorithm for the accuracy of K-value estimation.

### 4.3. Road Lateral Slope Estimation Validation Experiment

**Figure 10:**
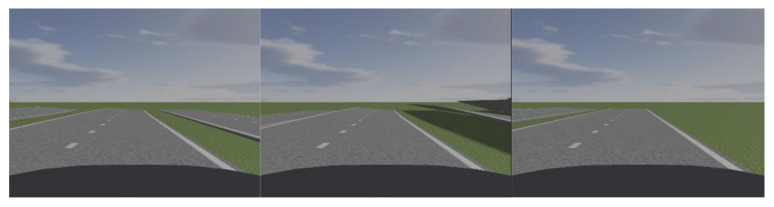
Schematic diagram of the experimental part of Prescan slope estimation.

**Figure 11:**
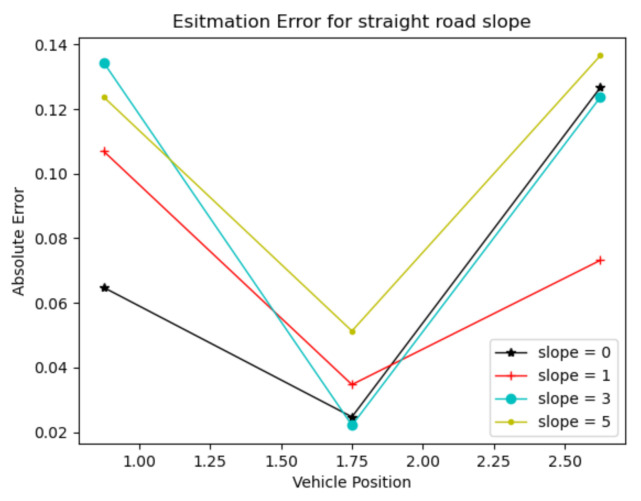
Graph of the results of estimating the lateral-slope angle of a straight road.

**Figure 12:**
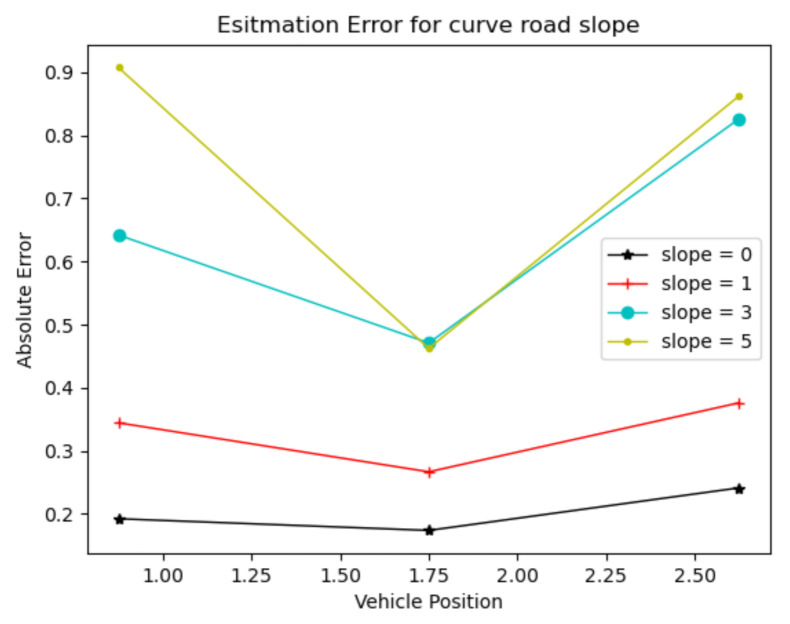
Estimation results of lateral-slope angle of curved road.

To verify the accuracy of the lateral-slope estimation algorithm, straight roads with lateral slopes of 0°, 1°, 3° and 5°, and curved roads with a radius of curvature of 500 m as shown in Figure 10, were designed, respectively. The vehicles were placed at three points on the lane quadrature for estimation experiments. Figure 11 shows the results of the straight-road slope estimation and Figure 12 shows the results of the curved-road slope estimation.

By analyzing the experimental results, we can see that the estimation error of the lateral slope of the straight road is within 0.15°, while the estimation error of the lateral slope of the curved road is within 1°. Thus, obviously, the estimation of the straight road is much more accurate than that of the curved one.

### 4.4. Slope Estimation Error Analysis

**Figure 13:**
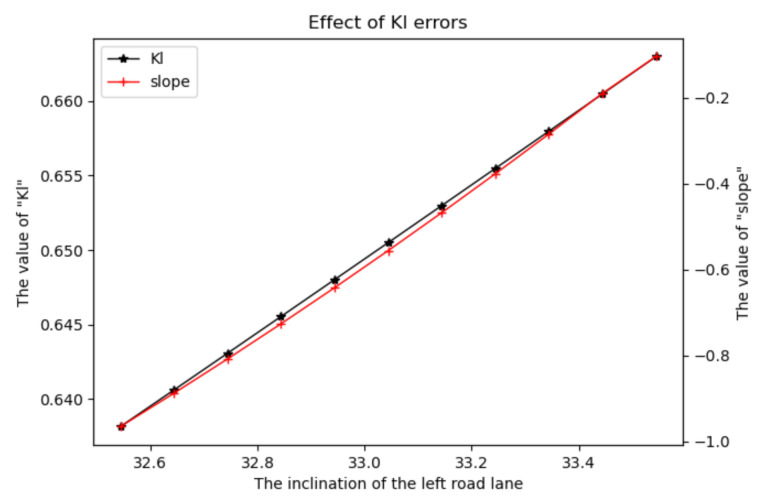
Error effect diagram of $K_{l}$.

In order to explain the reasons for the appearance of the above experimental results, this paper chooses to analyze from Equation (8). Taking a straight road with a lateral slope of 5° as an example, the left detection-fitted lane line is rotated by 1°. $d_{l}$ hardly changes during the lane line rotation, while the changes in $K_{l}$, as well as the road-slope estimation during this process, are shown in Figure 13.

Furthermore, by analyzing Figure 13, it can be seen that when the lane-line detection fitting is slightly deviated, it has a greater impact on the $K_{l}$ value, and also leads to the error divergence of the estimated road slope; that is, the error value of the road-slope estimation is strongly correlated with the lane-line detection fitting effect.

## 5. Conclusions

(1) In this paper, a simple analytical formula for the lateral slope of the road is derived with the help of the image perspective principle, which is related to only a few parameters and greatly reduces the complexity of the algorithm. The estimation results of the algorithm are more accurate for structured roads.

(2) By analyzing the slope estimation principle and conducting experimental verification, it is concluded that the accuracy of the slope estimation algorithm is strongly correlated with the detection of lane lines. By enhancing the detection accuracy of lane lines, the estimation error can be effectively reduced.

## Figures and Tables

**Table 1:** Experimental results of $d_{l}$ estimation.

$\mathrm{True}\;\mathrm{Value}\;d_{l}$	0.4375	0.8750	1.3125	1.7500	2.1875	2.6250	3.0625
Estimated value $D_{l}$	0.5248	0.8919	1.3212	1.7500	2.1867	2.6128	3.0225
Absolute error value	0.0873	0.0169	0.0087	0.0000	−0.0008	−0.0122	−0.0400

## Data Availability

Not applicable.
